# Lessons Learned from Telemonitoring in an Outpatient Bariatric Surgery Pathway—Secondary Outcomes of a Patient Preference Clinical Trial

**DOI:** 10.1007/s11695-023-06637-9

**Published:** 2023-07-07

**Authors:** Elisabeth S. van Ede, Jai Scheerhoorn, Friso M. J. F. Schonck, Jonna A. van der Stam, Marc P. Buise, Simon W. Nienhuijs, R. Arthur Bouwman

**Affiliations:** 1 Department of Anesthesiology, Catharina Hospital, Michelangelolaan 2, 5623 EJ Eindhoven, The Netherlands; 2 Department of Electrical Engineering, Signal Processing Systems, Eindhoven University of Technology, 5612 AP Eindhoven, The Netherlands; 3 Department of Surgery, Catharina Hospital, 5623 EJ Eindhoven, The Netherlands; 4 Department of Clinical Chemistry, Catharina Hospital, 5623 EJ Eindhoven, The Netherlands; 5 Department of Anesthesiology, Maastricht University Medical Center, 6229 HX Maastricht, The Netherlands

**Keywords:** Telemonitoring, Continuous and remote monitoring, Outpatient bariatric surgery, Clinical practice

## Abstract

**Background:**

Remote monitoring is increasingly used to support postoperative care. This study aimed to describe the lessons learned from the use of telemonitoring in an outpatient bariatric surgery pathway.

**Materials and Methods:**

Patients were assigned based on their preference to an intervention cohort of same-day discharge after bariatric surgery. In total, 102 patients were monitored continuously for 7 days using a wearable monitoring device with a Continuous and Remote Early Warning Score–based notification protocol (CREWS). Outcome measures included missing data, course of postoperative heart and respiration rate, false positive notification and specificity analysis, and vital sign assessment during teleconsultation.

**Results:**

In 14.7% of the patients, data for heart rate was missing for > 8 h. A day-night-rhythm of heart rate and respiration rate reappeared on average on postoperative day 2 with heart rate amplitude increasing after day 3. CREWS notification had a specificity of 98%. Of the 17 notifications, 70% was false positive. Half of them occurred between day 4 and 7 and were accompanied with surrounding reassuring values. Comparable postoperative complaints were encountered between patients with normal and deviated data.

**Conclusion:**

Telemonitoring after outpatient bariatric surgery is feasible. It supports clinical decisions, however does not replace nurse or physician care. Although infrequent, the false notification rate was high. We suggested additional contact may not be necessary when notifications occur after restoration of circadian rhythm or when surrounding reassuring vital signs are present. CREWS supports ruling out serious complications, what may reduce in-hospital re-evaluations. Following these lessons learned, increased patients’ comfort and decreased clinical workload could be expected.

**Trial Registration:**

ClinicalTrials.gov. Identifier: NCT04754893.

**Graphical Abstract:**

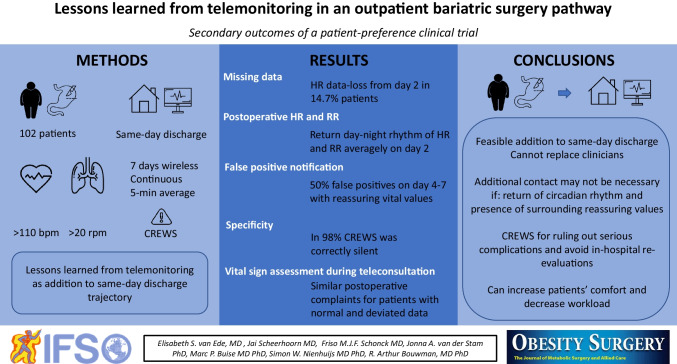

**Supplementary Information:**

The online version contains supplementary material available at 10.1007/s11695-023-06637-9.

## Background

Remote monitoring is increasingly used to support postoperative care. It is assumed continuous remote monitoring detects deterioration earlier by presence of deviating vital signs [1–3], and that ICU transfers or in-hospital deaths are reduced. [4, 5] However, barriers to practical usability are still mentioned challenging the implementation of remote monitoring for optimal clinical use [6–8].

Metabolic or bariatric surgery is an example of elective surgery mostly performed in high volume centers for the sake of quality. Increasing evidence suggests that bariatric surgery on an outpatient basis is possible and safe. [9] With globally increasing numbers of metabolic procedures every year, both same-day discharge and telemonitoring have the potential to improve healthcare efficiency in addition to health gains.

Continuous monitoring devices are more often being deployed in this field to support patients and clinicians remotely. Commonly cited barriers are connectivity issues and the risk of alarm fatigue. [3, 7, 10] The use of early warning scoring systems for the early detection of deterioration is also a matter of debate as is the fear of being overwhelmed or replaced by big data. [11] Some obstacles can be overcome by technical improvements, by exploring other detection procedures or even through a critical reassessment of their clinical relevance. This requires more detailed insights obtained from a clinical setting where remote monitoring has been applied prospectively.

Recently, we tested a new outpatient bariatric surgical pathway supported by remote monitoring prospectively during a patient preference–based clinical study. [12, 13] The aim of the current study is to describe the lessons learned from the use of remote monitoring as an addition to this trajectory and to optimize future use.

## Methods

Remote monitoring data from a prospective observational cohort was analyzed. This cohort was part of a recently conducted non-inferiority study based on patient preference. Herein, an outpatient recovery trajectory with remote monitoring (intervention group) was compared with an overnight hospital stay without monitoring (control group). [12, 13] Adult patients undergoing a primary bariatric procedure were eligible to participate. A total of 102 patients who were assigned to the intervention group based on their preference were included for analyses in the current study.

The surgical procedure was performed in the morning. Patients were discharged the same day in the evening if they were still willing to be discharged, the hemoglobin level decrease was ≤ 3.2 g/dl, and the spot-check measured heart rate (HR) was < 100 beats per minute (bpm). Care was supported by 7 days ongoing remote monitoring, starting on the day of surgery, using a portable monitoring device (Healthdot, Philips). Healthdot is a validated wearable, CE certified data logger supported with a Continuous and Remote Early Warning Score notification protocol (CREWS). [14, 15] The thresholds for generating a notification were set to 110 bpm for HR and 20 breaths per minute for respiratory rate (RR) based on previous research. [15] The wearable was used in addition to standard care in which the obtained vital signs and any notifications provided support to the clinicians for the assessment of the patient. Values were always assessed in the context of a patient’s individual situation. Postoperative follow-up was performed by a clinician. Patients who were discharged on the same day received teleconsultation the following morning (postoperative day 1). Vital signs (HR and RR) and any previous night’s notifications were reviewed. The following 5 days, the system was consulted every morning to assess vital parameters and any notifications of the past 24 h. The patients were instructed, in accordance with standard procedure, to contact the hospital at any time in case of questions, complaints, or problems. Also in this situation, the dashboard was consulted for additional information of vital functions.

The analysis included indicators that evaluate the added value of postoperative remote and continuous monitoring of vital functions: the amount of missing data > 2 h and > 8 h, evaluation of postoperative course HR and RR, the number of false positive notifications, the specificity of the CREWS notification system, and added value of vital signs monitoring during teleconsultations.

The onset of any complication was defined as the first sign present leading to the suspicion of a postoperative problem that ultimately required additional intervention classified as Clavien-Dindo 3a or b. A deviated postoperative course was defined as a suspicion that resulted in Clavien-Dindo ≤ 2. First signs could come from telephone contact by patients themselves or initiated by the hospital in response to the monitoring data. The postoperative complications and deviations were distinguished from whether a notification alerting the physician would have been essential. A notification was considered false positive if it was generated in all cases in which the notification was not essential. It was noted whether a clinician was concerned and whether any surrounding reassuring vital signs were present. The false positives were separated based on whether or not reassuring vital signs were available. Data was not reassuring if there had been a pre-notification trend increase or if vital signs had not yet returned to levels considered baseline for the patient. The lack of a day-night rhythm or fluctuation of vital signs was also reason for caution. Issues and treatments that emerged during telephone consultation on postoperative day 1 were collected and categorized based on characteristics of the corresponding vital signs: normal or abnormal. Vitals were considered abnormal if the physician assessed them as physiologically disturbed. For example, increased HR (> 100 bmp or increased compared to previous measurements) and/or increased RR (> 20 breaths per minute or increased compared to previous measurements).

All patients were included for analyses, including those who did not wear the device for the full 7 days after surgery. Group means of patient characteristics were assessed by using SPSS Statistic software version 25.0. All visualizations were made by using R 4.2.1. and R studio (version 22.07.02 build 576) software. The satisfaction score was assessed by an independent *t*-test and any difference considered statistically significant at a two-tailed *p*-value of less than 0.05. The median and interquartile ranges (IQR) of the percentage missing data and the occurrence of missing data for > 2 h or > 8 h were visualized. The activity level of the patients over 7 days was demonstrated as median and IQR. The median and IQR of vital measures of the postoperative HR and RR were plotted over 7 days, including the day of surgery. Examples of HR trends of patients with false positive notifications were displayed. A moving average of 1 h has been used for all figures that include HR and RR.

## Results

Patient characteristics are summarized in Table 1. In total, 144.540 5-min averaged data points measured in 16.767 h (average of 164.4 h and 6.9 days per patient) were available for analyses.

**Table 1:** Patient characteristics (*n* = 102)

Age (years)	40 ± 11.3
Female (%)	83 (81.4)
Body mass index kg/m^2^	43 ± 5.4
Hypertension – *n* (%)	25 (24.5)
Diabetes mellitus type II – *n* (%)	7 (7)
Gastroesophageal reflux disease – *n* (%)	14 (14)
Musculoskeletal pain – *n* (%)	28 (28)
Obstructive sleep apnea syndrome – *n* (%)	8 (8)
Surgery type – *n* (%)	
- Sleeve gastrectomy - Roux-en-Y gastric bypass	54 (53) 48 (47)
Surgery duration – minutes	53 ± 17.5
Overall satisfaction score telemonitoring	8.0 ± 1.6

The patients in this cohort had a preference for same-day discharge supported by remote monitoring. These patients (*n* = 102) were equally satisfied with the care received compared to the patients who opted for an overnight stay without remote monitoring (*n* = 100) (8.0 ± 1.6 versus 8.0 ± 1.4 respectively *p* = 0.86, 95% CI − 0.4 to 0.5).

### Amount of Clinically Relevant Missing Data

**Fig. 1:**
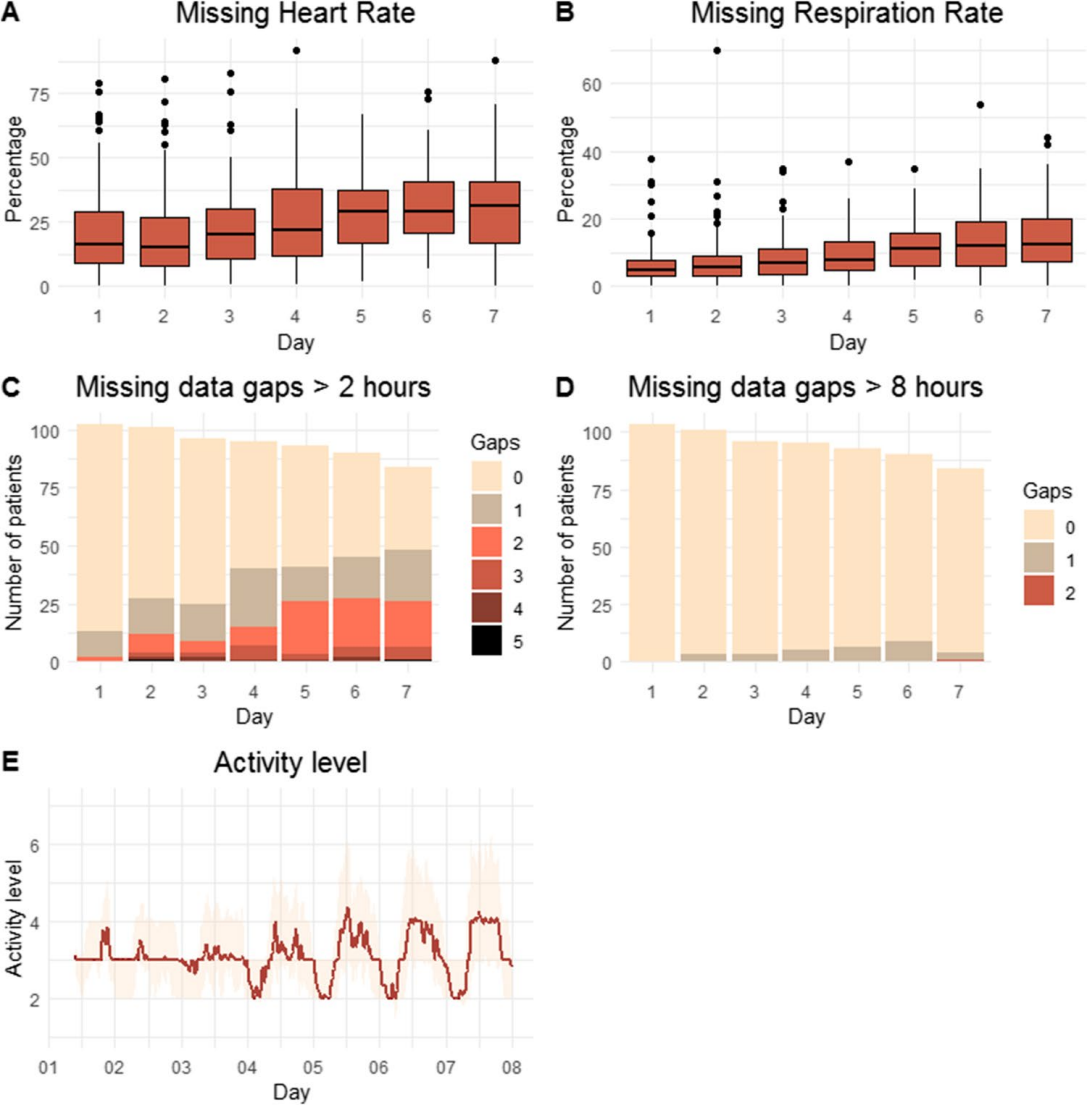
Amount of missing data for heart rate. Missing data heart rate (**A**) and respiration rate (**B**), activity level (**C**), missing data gaps > 2 h (**D**) and missing data gaps > 8 h (**E**). X-axis: days postoperative, Y-axis: **A** and **B** percentage and **C** activity level, and **D** and **E** activity level

Of the 102 patients in total, 18 (17.6%) patients wore Healthdot for less than 7 days. In 7 (6.9%) of them, this was shorter than 4 days (1–3 days). The amount of missing data increased with time after surgery, and was lower for RR then for HR (Fig. 1A and B). In 73 (71.5%) patients, there were missing data of HR > 2 h during the 7 days. This occurred on the day of the surgery itself in 13 patients, in 11 patients 1 time and 2 patients 2 times (Fig. 1C). In 15 patients (14.7%) of those with missing data of HR > 2 h, the interruption lasted longer than 8 h. This never happened on the day of the surgery itself however from day 2 or 3 (Fig. 1D). Also, during these gaps, the measurement for RR remained undisturbed. The day-time activity level increased and the night-time activity decreased with time (Fig. 1E).

### Course of the Postoperative Heartrate

**Fig. 2:**
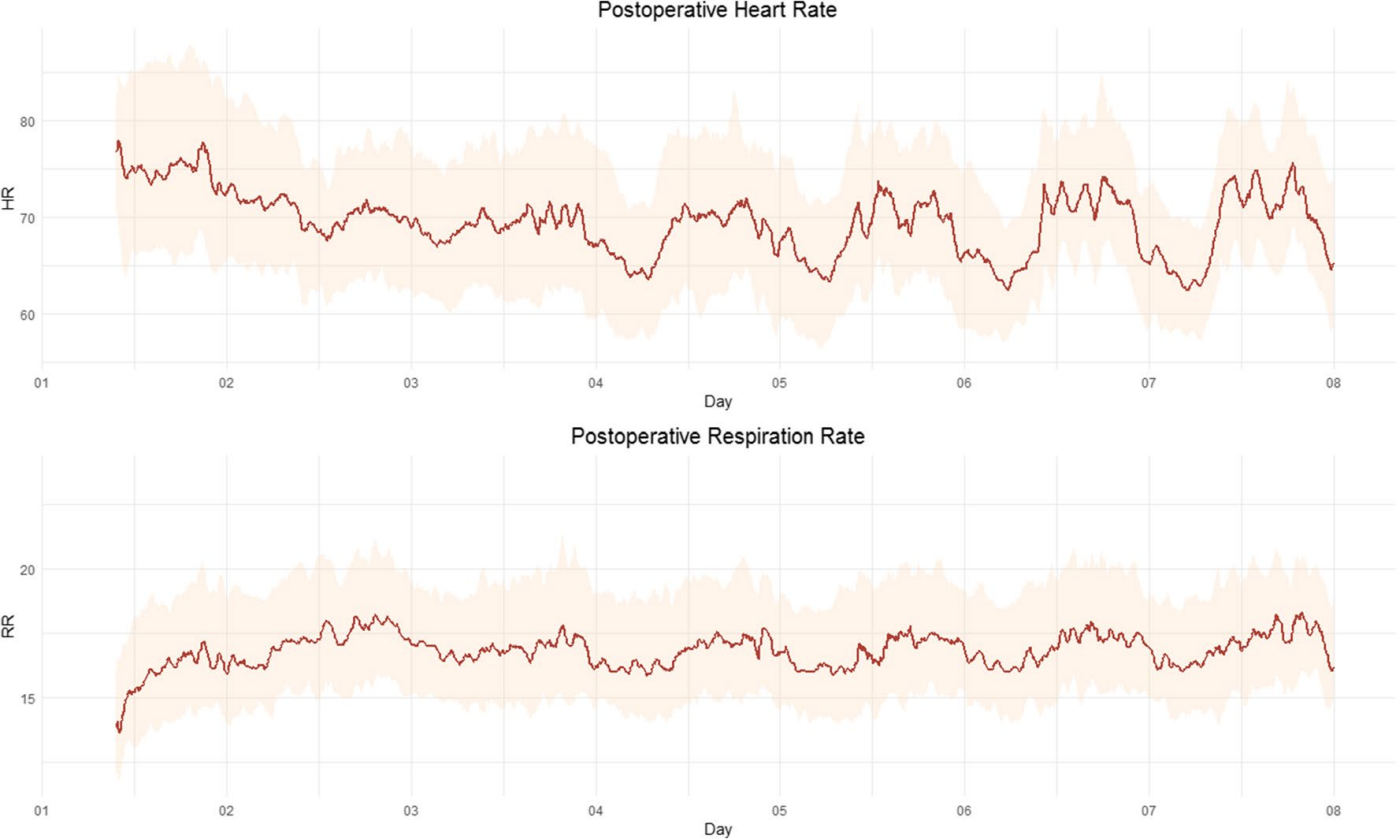
Postoperative course heart rate and respiration rate. X-axis: days postoperative, Y-axis: heart rate in beats per minute. Red line: median heart rate (upper) and respiration rate (lower); the cloud around it represents the IQR

Figure 2 shows the course of postoperative HR and RR during the first 7 days. In the first 8 h, median HR is at its highest, after which a decrease follows. This decrease stabilizes around the second post-surgery day in the afternoon. From then on, a day-night rhythm of the HR can be observed. The amplitude of the day-time and night-time HR increases after day 3, due to a decrease in nocturnal resting HR and a global increase in peak day-time HR. For RR, the rate increases from surgery until the morning on day 2. Together with a further increase on day 2, a day-night rhythm seems to reappear which is earlier than for HR. Also, the course of the amplitude is different as it does not increase with time after surgery.

### False Positive Notifications

See Table 2 for a complete overview of the events.

**Table 2:** Overview postoperative outcomes. *CD* Clavien-Dindo classification

*n* = 102	Outcomes			
	Notification not essential		Notification crucial	
	Uncomplicated	Deviation	Deviation	Complicated
*n* patients	95	1 superficial wound infection (CD2) 1 readmission (CD1)	1 bleeding (CD2)	4 bleeding (1 × CD3a, 3 × CD3b)

**Fig. 3:**
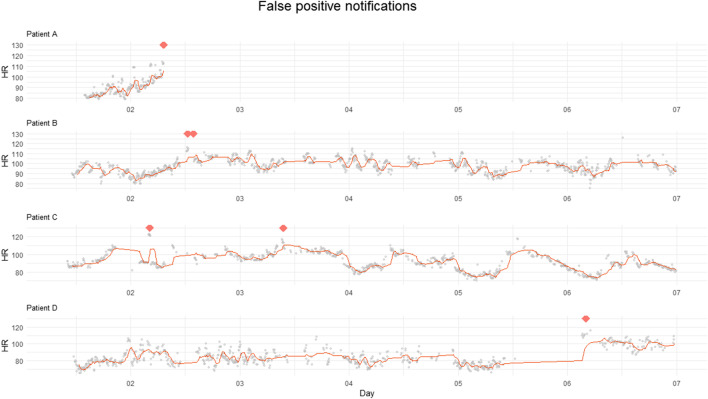
Heart rate trend in patients with false positive notifications. Four different patients with false positive notifications in who contact seemed necessary. HR = heart rate, X-axis = days postoperative, Y-axis = HR in bpm, solid line = median HR, dots = outliers, diamond shape = notification

**Fig. 4:**
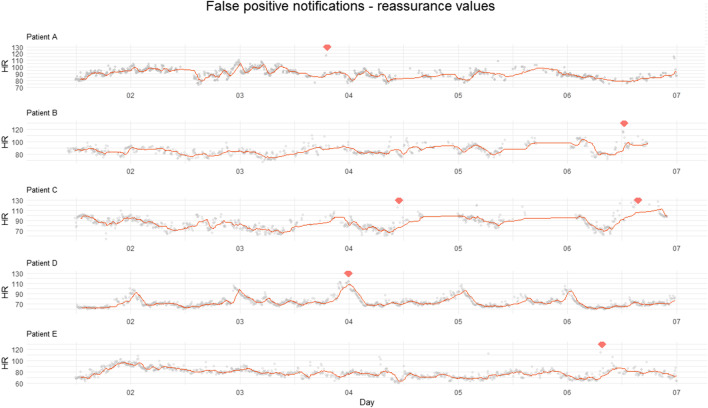
Heart rate trend in patients with false positive notifications with reassuring values. Five different patients with false positive notifications in who contact seemed not necessary. HR = heart rate, X-axis = days postoperative, Y-axis = HR in bpm, solid line = median HR, dots = outliers, diamond shape = notification

In total, 17 notifications were produced in 13 patients. Of these 17 notifications, 70% (12 alarms) were positive while no complication occurred. These false positive notifications are shown in Figs. 3 and 4.

In Fig. 3, patient A was contacted because it seemed that the connection was lost after the notification. Patient B was experiencing severe pain for which oral opioids were prescribed. Patient C was mobilizing, and patient D was contacted because there was no apparent return of circadian rhythm, along with a large gap in the data followed by a strong increase in HR that triggered a notification. The patients from Fig. 4 were also contacted; however, the chance that a complication was present was estimated to be very low in advance. None of the patients showed clinical signs of internal bleeding or sepsis.

Notifications without reassuring vitals mostly occurred around day 2–3, notifications with reassuring vitals around day 4–7.

### Specificity of the CREWS Notification System

The number of patients generating > 1 notification per day was 4 (4%). CREWS was completely silent in 87% of the patients (89 patients out of 102). In 98% of the cases (87 of the 89 patients without a notification), CREWS was correctly silenced.

### Vital Sign Assessment During Teleconsultation

Comparable complaints were encountered on postoperative day 1 between patients with normal and deviated data (Table 3).

**Table 3:** Vital sign assessments on scheduled teleconsultation on postoperative day 1 (*n* = 66). *CD* Clavien-Dindo

Normal data (60)	Situation (*n*)	Action (*n*)
No issues (56)	**-**	-
Issues (4)	Pain (1)	Extra teleconsult requested by patient
	Nausea (2)	Medication (2) + extra teleconsult requested by 1 patient
	Pale, clammy skin, pain, nausea, rectal blood loss—not feeling well (1)	Reassessment in hospital (CD3a readmission)
Deviated data (6)	Situation (*n*)	Action
Elevated HR	Nausea and vomit old blood cloths—not feeling well (1)	Reassessment in hospital (CD1 readmission)
Elevated RR	Pain and nausea (3)	Medication
Elevated HR and RR	Pain (1)	Medication and extra teleconsult
Notification day 1 04:11 am	No issues (1)	Reassess monitoring data: 9:00a.m. vitals recovered

## Discussion

Our previous study described the feasibility of outpatient bariatric surgery supported by remote vital parameters monitoring. [12] The current addition describes the lessons learned out of this process. Technical performances, characteristics of Healthdot, and clinical consequences were reviewed in 102 patients.

Signs of postoperative bleeding are often revealed between 8 and 24 h postoperative. After this period, the risk of bleeding decreases and symptoms of anastomotic leakage may occur with vital signs usually changing steadily. [16] Our results showed that approximately 15% of monitored patients missed HR data for periods > 8 h, while RR data remained uninterrupted. This happened mostly from the second postoperative day. This proportion may seem high, however could be considered clinically irrelevant as it is beyond most risky period and still providing more insight than current practices without any monitoring.

As the amount of missing data on the first day and night was low, the ability to detect postoperative complications was not compromised. Moreover, the measurements are accurate and have a higher density than information available in hospitals where vital signs are measured manually at least every 8–12 h. [14, 17] This benefits deterioration detection in case of missing data or for the assessment of a notification. These so-called reassuring measurements include resting HR (especially at night) [18] and undisturbed RR measurements. This combined to the retrospective assessment of vital signs every 24 h, made detection of anastomotic leakage signs still possible.

It was observed the amount of missing data increased with time after surgery. This could be explained by increase in activity level during the day, which may be a sign of recovery. [7] Missing day-time data in the home situation may therefore not be a cause of concern; however, it does require attention if this already occurs in the hospital in a non-mobilizing patient, or shortly after discharge. Healthdot is an accelerometer and therefore sensitive to movement artifacts, which could lead to missing data from patients with severe pain or delirium. A similar amount of data loss was also found by Breteler et al. [7] They suggested reducing the measurement frequency to minimize the number of warnings due to missing data. However, our findings indicate that missing data from a certain point in time could be ignored provided reassuring measurements are present, which argues against phasing out the measurement frequency.

The elevated postoperative HR followed by a decrease in the current setting has been previously observed by other researchers, [7] as has the return of a day and night rhythm. [20, 21] After surgery and general anesthesia, the circadian rhythm of various endogenous rhythms is disturbed including HR. [22] This may be caused by the anesthesia, but also with the operation or hospitalization itself. [23] In ICU and COVID-19 patients on the generic ward, a smaller peak nadir amplitude of the HR, a disrupted circadian rhythm, was seen in deceased patients compared to those who recovered. [20, 21] It could be suggested that an increasing amplitude of the day-night rhythm after surgery or illness may indicate the presence of recovery [20, 21, 24].

In the present study, the CREWS system was used. [15] Of the 17 notifications put forward by this system, 70% was false positive. Half of them occurred between postoperative day 4–7 and were accompanied with surrounding reassuring vital values. As reassurance vital values help to distinguish between internal pathology and normal exercise physiology, this result might suggest that in patients with a notification after postoperative day 3 who have reassuring surrounding vital signs or whose amplitude of the day and night rhythm of the HR has increased, no additional contact would be necessary. Elimination of such alarms (*n* = 6) halves the false positive rate from 70 to 35%. Completely abolishing notification use has been discussed by Leenen et al. [25] However, as the trends of the patients were visualized in hospital six times a day, this outcome cannot yet be translated to a home monitoring process. In addition, the specificity of the system was 98%, which is comparable to previous results. [15] The reporting system can facilitate ruling out clinical deterioration and prevent hospital reassessments. In addition to trend assessment of vital values, the absence of a notification can be detected with a quick look which may lower workload safely. These positive findings that were encountered in this study, and that would be absent without telemonitoring, are anecdotal. However, this advantage is expected to grow with increased use. The complaints encountered on the first day after surgery in this study were comparable between patients with normal and deviated data. Other factors, such as clinical history, physical examination, and lab values or biomarkers, remain important for clinical decision-making. [26, 27] This is in line with how we currently use remote monitoring for postoperative patients. It supports the regular care path where values are interpreted exclusively in the context of the individual patient.

This study was limited by a low complication rate. Also, no data on cases of anastomotic leakage was available as mainly postoperative bleeding occurred. Since implementation with continuously monitored data requires confidence in assessing data and trends, it can take a little longer to gain experience. [6, 28] The next step is large-scale implementation to realize this and to answer follow-up research questions such as detailed mapping of changes in workload, cost-effectiveness analysis, and evaluation of patient morbidity or mortality. Other ways of monitor optimization to detect deterioration can be explored. For example, adjusting or personalizing alarm limits and reassurance time, [8, 15, 29, 30] but also algorithms, [31] predictive algorithms [32], or trend analysis including circadian rhythm seem promising techniques [20, 33].

## Conclusion

Our results indicate that telemonitoring after outpatient bariatric surgery is feasible and supports clinicians, but cannot replace nurse or physician care in its current form. The false notification rate is high; however, it can be suggested that no additional contact is necessary when a notification has been judged by clinicians to occur after restoration of circadian rhythm or when surrounding reassuring vital signs are present. Moreover, CREWS can be used to rule out serious complications and avoid re-evaluations in the hospital. Following these lessons learned, increased patients’ comfort and decreased clinical workload could be expected.

## Supplementary Information

Below is the link to the electronic supplementary material.

Supplementary file1 (DOCX 275 KB)
